# Species Composition and Toxigenic Potential of *Fusarium* Isolates Causing Fruit Rot of Sweet Pepper in China

**DOI:** 10.3390/toxins11120690

**Published:** 2019-11-24

**Authors:** Jianhua Wang, Shuangxia Wang, Zhiyong Zhao, Shanhai Lin, François Van Hove, Aibo Wu

**Affiliations:** 1 Institute for Agri-food Standards and Testing Technology, Shanghai Academy of Agricultural Sciences, 1000 Jinqi Road, Shanghai 201403, China; wangjianhua@saas.sh.cn (J.W.); zhaozhiyong@saas.sh.cn (Z.Z.); linshanhai@gxaas.net (S.L.); 2 SIBS-UGENT-SJTU Joint Laboratory of Mycotoxin Research, CAS Key Laboratory of Nutrition, Metabolism and Food Safety, Shanghai Institute of Nutrition and Health, Shanghai Institutes for Biological Sciences, Chinese Academy of Sciences, University of Chinese Academy of Sciences, Shanghai 200000, China; shuangxiawang@163.com; 3 Sugarcane Research Institute, Guangxi Academy of Agricultural Sciences, Nanning 530007, China; 4 Mycothèque de l’UCL catholique de Louvain (BCCMTM/MUCL), Applied Microbiology (ELIM), Earth and Life Institute (ELI), Université catholique de Louvain (UCL), B-1348 Louvain-la-Neuve, Belgium; francois.vanhove@uclouvain.be

**Keywords:** *Fusarium* species, mycotoxin, toxigenic profile, mycotoxin migration, sweet pepper, fungal disease

## Abstract

Apart from causing serious yield losses, various kinds of mycotoxins may be accumulated in plant tissues infected by *Fusarium* strains. Fusarium mycotoxin contamination is one of the most important concerns in the food safety field nowadays. However, limited information on the causal agents, etiology, and mycotoxin production of this disease is available on pepper in China. This research was conducted to identify the *Fusarium* species causing pepper fruit rot and analyze their toxigenic potential in China. Forty-two *Fusarium* strains obtained from diseased pepper from six provinces were identified as *F. equiseti* (27 strains), *F. solani* (10 strains), *F. fujikuroi* (five strains). This is the first report of *F. equiseti*, *F. solani* and *F. fujikuroi* associated with pepper fruit rot in China, which revealed that the population structure of *Fusarium* species in this study was quite different from those surveyed in other countries, such as Canada and Belgium. The mycotoxin production capabilities were assessed using a well-established liquid chromatography mass spectrometry method. Out of the thirty-six target mycotoxins, fumonisins B_1_ and B_2_, fusaric acid, beauvericin, moniliformin, and nivalenol were detected in pepper tissues. Furthermore, some mycotoxins were found in non-colonized parts of sweet pepper fruit, implying migration from colonized to non-colonized parts of pepper tissues, which implied the risk of mycotoxin contamination in non-infected parts of food products.

## 1. Introduction

Sweet pepper or bell pepper (*Capsicum annuum* L.) is highly appreciated in the fresh vegetable markets worldwide due to its unique taste, aromas, and the multiple culinary uses. It represents one of the most important vegetables for the high content of phytochemicals, such as ascorbic acid and soluble phenols [1,2], having potential positive effects on human health. For example, ascorbic acid is an essential dietary nutrient in the human body with its vital biological function as an antioxidant. Sweet pepper is an economically important vegetable crop and widely used for direct consumption and manufacturing of sauce worldwide, where the global production reached 34.6 million tons of fresh fruit and 3.5 million tons of dried pods [3]. China is the largest producer and exporter of sweet pepper in the world, with a total fresh pepper production per year of almost 23 million tons. Sweet pepper is often grown in commercial greenhouses, which is favorable for the growth and survival of phytopathogenic fungi and the infection of pepper plants [4]. Thus, the disease poses a serious limitation to pepper cultivation, resulting in yield reduction or complete crop loss, as reported previously in the literature [4,5,6].

Several fungal diseases have caused economic losses in sweet pepper production in Canada, United Kingdom, and Belgium [4,7,8]. *Fusarium* infection on the stem- and blossom-end of pepper fruit caused by *F. solani* was first reported in Ontario and British Columbia in 1991 and caused approximately 5% fruit-yield loss [8,9,10]. Since the 1990s, this rot disease has been an increasing problem in pepper production in both Europe and North America [7,11,12]. More seriously, a severe outbreak of this disease resulting in a 50% yield loss in a greenhouse was reported in 1990 in Ontario [13].

In addition to external fruit rot, internal rot of sweet pepper fruit is also a big problem in pepper production [14]. In contrast to the external pepper fruit rot, the fruit is infected internally by a fungus. Unless severely infected and rotten, most infected fruits are difficult to cull before delivery to the market as the symptoms are not readily visible [4,8,10]. A comprehensive histopathology analysis performed by Yang et al. [10] indicated that internal fruit rot of greenhouse sweet pepper caused by *F. lactis* was initiated through the infection of the stigma and style during anthesis. Symptomless seed infection may contribute to disease spread, and air and insects also play an important role as intermediates in fungal spores spreading [15]. Based on the surveys to date, *F. lactis* is the principal causal agent of internal fruit rot of pepper, although the closely related *Fusarium* species belonging to the *Fusarium fujikuroi* species complex (FFSC), such as *F. proliferatum*, *F. subglutinans,* and *F. verticillioides*, have also been implicated in this disease [4,6,8].

**Figure 1:**
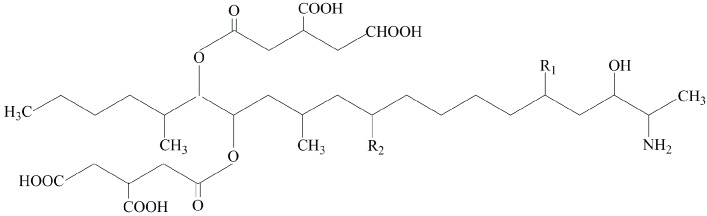
Structural formulas of B-series fumonisins.

Apart from causing significant yield losses, *Fusarium* species can produce fumonisins (FBs), trichothecenes (TCs), zearalenone (ZEN), and other mycotoxins in infected plant tissues which are harmful to consumers. For example, B-series fumonisins (Figure 1), which are mainly produced by *Fusarium* species from FFSC, are the most frequently detected mycotoxins in maize, and are involved in animal and human diseases by interfering with sphingolipid metabolism. Moreover, fumonisins have been associated epidemiologically with esophageal cancer in humans in some regions of the world [16,17]. On the basis of available toxicological evidence, the International Agency for Research on Cancer (IARC) has classified fumonisins as possibly carcinogenic (Group 2B) [18]. Mycotoxin contamination in grains, vegetables, and fruit poses a serious threat to food safety.

Mycotoxins can diffuse into tissues surrounding the pathogen-infected site. The water content of fresh sweet pepper is usually higher than 90%, which contributes to the dissolution of polar compounds produced by pathogens in pepper tissues. Fumonisins, fusaric acid (FA), and moniliformin (MON) are the most common mycotoxin contaminants produced by *Fusarium* species, and migration of some of these toxins has been reported in pepper and fruit previously [19,20]. A migration study of beauvericin (BEA) and fumonisins was reported by Monbaliu et al. [19], and the results indicated the migration of fumonisins into healthy parts of the sweet pepper, while beauvericin was not detected in the tissue surrounding the lesion. Mycotoxin contaminations originating from mycotoxin-producing *Fusarium* species in pepper should be considered as an importantly sensitive food safety concern. Therefore, it is of great necessity to assess the types and levels of mycotoxin contamination in pepper and its derived products [4].

Effective management of mycotoxin contamination in sweet pepper relies on the control of the fungal infection and requires a better understanding of *Fusarium* biology and epidemiology. To our best knowledge, there is little information on the causal agent, etiology, toxigenic potential, and geographic distribution of the *Fusarium* species involved in pepper disease in China. The objectives of this current work were: (i) To isolate and identify the causal organism from *Fusarium* genus on sweet pepper in China; (ii) to assess the potential mycotoxin profiling of the fungal pathogens; (iii) to estimate the migration behavior of mycotoxins in sweet pepper.

## 2. Results and Discussion

### 2.1. Isolation and Identification of Fusarium Isolates

Sweet pepper fruits were sampled from Hainan, Heilongjiang, Hunan, Shanghai, Shandong, and Zhejiang provinces in China. Following isolation from diseased pepper tissues, a total of forty-two single-spore isolates were obtained and identified as belonging to *Fusarium* genus by morphology. Two of theses strains were isolated from Hainan, one strain was isolated from Heilongjiang, five strains were isolated from Hunan, twenty-one strains were isolated from Shanghai, three strains were isolated from Shandong, and ten strains were isolated from Zhejiang (Table 1).

The purified isolates were first identified to species with morphological characteristics [21], and this was subsequently confirmed by nucleotide sequences analysis of the translation elongation factor 1-α (*TEF*-1α) genes with partial of representative strains. Of the forty-two strains, twenty-seven were identified as *F. equiseti*, ten as *F. solani*, and five as *F. fujikuroi* (Table 1). Previously, a new disease on pepper caused by *F. concentricum* was reported by our group, and the strain MUCL54697 was isolated from Hunan province, China [22].

Eleven representative strains belonging to different species were selected for *TEF-1α* gene sequencing to further confirm the morphological identification results as described before [23,24,25]. Obtained sequences were subjected to alignment analysis using the network service tool BLASTn of the National Center for Biotechnology Information (NCBI) database. The sequence analysis of the portion of the *TEF-1α* genes of representative strains confirmed that all the strains belonged to *Fusarium* genus. The results indicated that nucleotide sequences of three strains (Q12002, Q12003 and Q12005) showed the highest identity (>99%) with the sequence of *F. equiseti*, three (Q12029, Q12030 and Q12034) showed the highest identity (>99%) with the sequence of *F. solani,* and five (Q12038-Q12042) showed the highest identity (>99%) with the sequence of *F. fujikuroi* in the NCBI database. The molecular identification results were all identical with the morphological results. The *TEF-1α* gene sequences generated in this study were deposited in GenBank, NCBI (http://www.ncbi.nlm.nih.gov/genbank/), under accession numbers KF208617–KF208627, which is presented in Table 1.

Among the forty-two *Fusarium* strains, all the ten *F. solani* (23.81%) strains were isolated from Shanghai, and the five *F. fujikuroi* (11.90%) strains were isolated from Hainan (one strain), Hunan (two strains), and Zhejiang (two strains). *F. equiseti* was the only species isolated from all six sampled provinces in China, and accounting for 64.29% of all *Fusarium* strains isolated (Table 1). According to our survey, *F. equiseti* was the predominant pathogen of sweet pepper fruit rot in China.

*Fusarium* strains were isolated from both external and internal rotten pepper fruits in this study. Of the forty-two strains, thirty-four were isolated from external rotten pepper fruits and eight strains were isolated from internal rotten pepper fruits (Table 1). For the twenty-seven *F. equiseti* strains, twenty-two were associated with external rot disease of pepper fruits and five (Q12002, Q12005, Q12008, Q12018, and Q12022) were isolated from internal rotten pepper fruits. For the ten *F. solani* and five *F. fujikuroi*, two (Q12034 and Q12035) and one strain (Q12041) were isolated from internal rotten pepper fruit, respectively. As such, *F. equiseti*, *F. solani*, and *F. fujikuroi* were the causal agents of external fruit rot of pepper in China, with *F. equiseti* being the predominant, while *F. equiseti*, *F. solani*, and *F. fujikuroi* were associated with internal fruit rot of pepper, with *F. equiseti* being the predominant. To our best knowledge, this is the first report of *F. fujikuroi*, *F. equiseti,* and *F. solani* associated with external and internal pepper fruit rot in China.

*F. solani* was reported as the predominant causal agent of external fruit rot of pepper in Europe and North America [7,8,9,10,11,12,13], which is different from China. However, it worth noting that *F. solani* is a common pathogen causing pepper fruit rot worldwide. *F. solani*, *F. lactis*, *F. proliferatum*, *F. subglutinans*, and *F. verticillioides* were reported to cause external or internal pepper fruit rot in Belgium, Canada, and United Kingdom, with *F. lactis* being the principal one [4,6,8]. In this study, *F. equiseti*, *F. solani*, and *F. fujikuroi* were found to be associated with internal pepper fruit rot, and with *F. equiseti* being the predominant. In light of the above, it is obvious that the population structure of *Fusarium* species associated with pepper fruit rot (external or internal) in China is quite different from those in surveys from Canada and Belgium [4,8,10]. Although the underlying factors for species distribution are unknown, climatic conditions (such as the annual temperature weather and humidity), hosts and their rotation, and adaptive evolution have been reported to influence the distribution of *Fusarium* species [14,26,27,28]. In view of the population genetic diversity and dispersal difference of the *Fusarium* pathogens, procedures for effective management of these pathogens on pepper are urgently needed.

### 2.2. Molecular Phylogenetics

**Figure 2:**
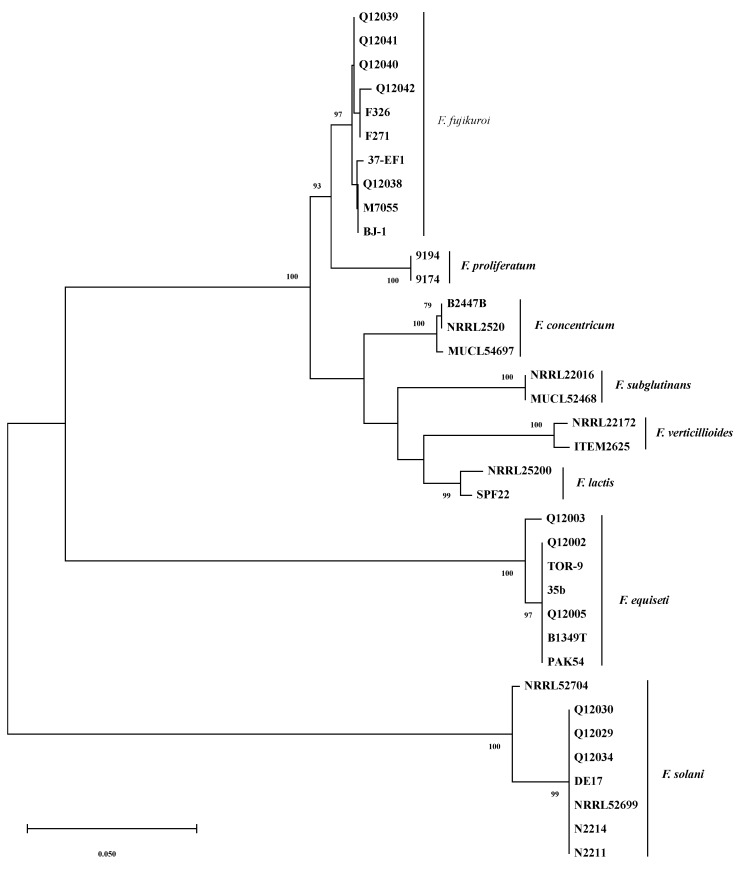
Phylogenetic tree inferred from alignments of *TEF-1α* sequences of *Fusarium* species by the Neighbor-Joining method with program MEGA 5.10. The numbers beside branches are the percentages of congruent clusters in 1000 bootstrap trials. Bootstrap values higher than 75% are shown.

Phylogenetic analyses were conducted on partial sequences of *TEF-1α* genes. Figure 2 shows the phylogenetic tree constructed with MEGA 5.10 [29]. As several *Fusarium* species have been reported to be the causal agents of pepper fruit rot, in addition to the nucleotide sequences obtained in this study (Table 1), corresponding sequences available in GenBank for the strains belonging to *F. concentricum*, *F. equiseti*, *F. fujikuroi*, *F. lactis*, *F. proliferatum*, *F. solani*, *F. subglutinans*, and *F. verticillioides* were retrieved and served as references.

As shown in Figure 2, bootstrap analyses of the *TEF-1α* gene partial sequences clearly separated the thirty-six *Fusarium* isolates into three major clades with a bootstrap value of 100%; the *F. equiseti* clade (seven isolates), the *F. solani* clade (eight isolates), and the third clade that was composed of the remaining twenty-one isolates belonging to *F. concentricum*, *F. fujikuroi*, *F. lactis*, *F. proliferatum*, *F. subglutinans*, and *F. verticillioides*. The six *Fusarium* species mentioned above in the third clade are all from the *F. fujikuroi* species complex (FFSC) [30], and they are relatively closer species among the species analyzed.

It is obvious that six distinct subclades were formed with >97% bootstrap support in the third clade which separated the twenty-one isolates to *F. concentricum* (three isolates), *F. fujikuroi* (ten isolates), *F. lactis* (two isolates), *F. proliferatum* (two isolates), *F. subglutinans* (two isolates), and *F. verticillioides* (two isolates) (Figure 2). Thus, the *TEF-1α* sequences can efficiently differentiate these *Fusarium* species. The *TEF-1α* sequence BLASTn and phylogenetic analysis results strongly supported the identification results of *Fusarium* strains isolated.

### 2.3. Multi-Mycotoxin Analysis in Pepper Fruit

**Figure 3:**
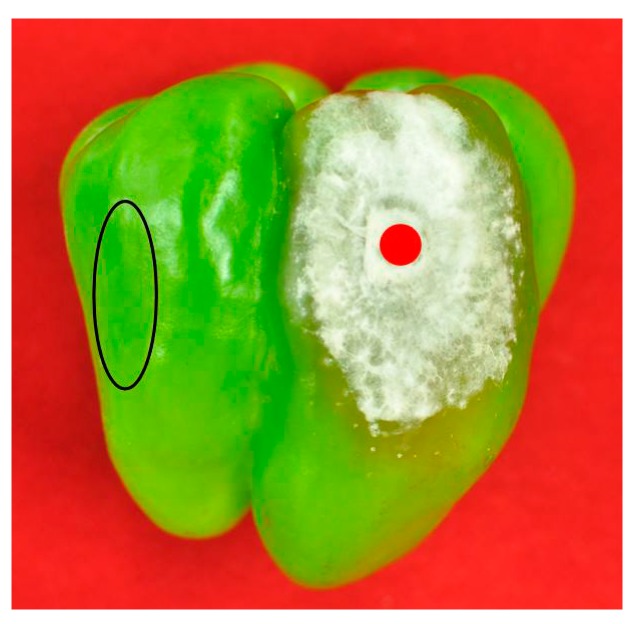
Inoculated pepper with the indication of inoculation site (red dot) and healthy site (black oval) for mycotoxin analysis.

For large-scale screening of mycotoxin production capabilities in pepper by the sampled *Fusarium* isolates, the well-established multi-component LC-MS/MS method was used for scanning of the 36 mycotoxins reported in agricultural products. In this multi-mycotoxin analysis, two *F. equiseti* isolates (Q12002 and Q12004), three *F. solani* isolates (Q12029, Q12034 and Q12037), four *F. fujikuroi* isolates (Q12038, Q12039, Q12040 and Q12041), and the *F. concentricum* strain MUCL54697 [22] were selected to do the inoculation experiment and assess their mycotoxin production in different sites of sweet pepper fruits (Figure 3).

**Figure 4:**
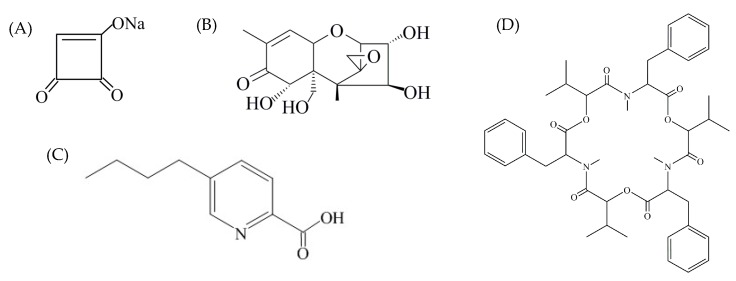
Structural formulas of moniliformin (**A**), nivalenol (**B**), fusariuc acid (**C**), and beauvericin (**D**).

Mycotoxin detection results from lesions (Figure 3, red dot) were used to do the mycotoxin profile analysis, and the results from the healthy parts (Figure 3, black oval) were used for the migration study. Mycotoxin profiles of the ten strains selected were summarized in Table 2. The multiple mycotoxin analysis resulted in the detection of BEA, FA, fumonisin B_1_ (FB_1_), fumonisin B_2_ (FB_2_), MON, and nivalenol (NIV) in pepper tissues (Figure 1 and Figure 4). In one out of the ten isolates inoculated, none of the thirty-six mycotoxins investigated was detected. FA was the most frequently detected metabolite which was produced by nine isolates in concentrations that greatly varied from 41.44 to 10,662.36 μg/kg. BEA was produced by eight isolates with average concentrations varying between 5.20 and 1019.60 μg/kg. Both FB_1_ and FB_2_ were produced by the same four isolates ranging from 43.64 to 39,326.60 μg/kg and 26.96 to 3734.16 μg/kg, respectively. MON was produced by five isolates in greatly varying amounts (35.80–2439.48 μg/kg), while NIV was produced by only one isolate at a concentration of 184.16 μg/kg.

As shown in Table 2, significantly different toxigenic profiles were observed among different species in inoculated pepper fruits. For the investigated *Fusarium* species, the most abundant of toxic metabolites were produced by *F. fujikuroi*, and all the four *F. fujikuroi* isolates can produce BEA, FA, FB_1_, FB_2_, and MON. With regard to the individual mycotoxin, relatively higher contents of FB_1_ were generated compared to FB_2_ by the same strain, which were consistent with the previously reported studies [31,32]. The ratios between the two fumonisins for individual *F. fujikuroi* strain were in the range of 1.05–1.83 in pepper lesions. Meanwhile, significant differences in fumonisin production capacities of *F. fujikuroi* strains were observed. For example, strain Q12040 produced FB_1_ and FB_2_ in concentrations of 43.64, 26.96 μg/kg, respectively, while large amounts of FB_1_ and FB_2_ were produced by isolate Q12041 (3926.60, 3734.16 μg/kg, respectively) which were about 90 and 139 times as higher than those produced by Q12040 under the same conditions. Note that similar or higher amounts of FA and MON were produced by the four *F. fujikuroi* strains when compared with FBs. For example, the amount of FA is higher than the total fumonisins (FB_1_ + FB_2_) produced by three out of four isolates. Strain Q12002 produced NIV, a kind of type B trichothecene, at a concentration of 184.16 μg/kg, while trichothecenes were not observed with the other strain Q12004. These two *F. equiseti* isolates showed a considerable intraspecies variation in profiles of trichothecene production, similar to results reported by Hestbjerg et al. [33]. Similarly, intraspecies variation in toxigenic profiles was also observed in *F. solani*. Regarding the three *F. solani* strains, only FA was produced by strain Q12029, compared to BEA and FA (94.84, 78.98 μg/kg, respectively) that were produced by strain Q12034, while none of the thirty-six mycotoxins were detected with strain Q12037.

Large variation among strains, both in terms of their toxigenic profiles and the quantity of mycotoxins produced in pepper, was found in this study. Based on the mycotoxin profile results, it could be concluded that interspecies toxigenic profile variation appears to be a species-specific characteristic, while the intraspecies quantity variation appears to be a strain-specific characteristic.

The results of the migration study are summarized in Table 2. Among the mycotoxins detected in this study, diffusion phenomenon of FA, FB_1_, FB_2_, and MON from a moldy area to healthy tissues was observed in pepper fruit, while no detectable BEA and NIV were found in unaffected parts.

As shown in Table 2, FA was the most frequently detected mycotoxin in unaffected parts, with concentrations varying from 13.80 to 874.04 μg/kg. As a phytotoxin [34], the ratios of FA detected from lesions and healthy parts were in the range of 5.54–51.87. FB_1_ was detected in unaffected parts with concentrations varying from 41.08 to 183.04 μg/kg. The ratios of FB_1_ detected from lesions and healthy parts were in the range of 21.45–37.95. FB_2_ was detected in unaffected parts with concentrations varying from 24.30 to 144.92 μg/kg and the ratios of FB_2_ detected from lesions and healthy parts were similar to FB_1_ (25.77–37.58). MON was detected in unaffected parts with concentrations varying from 172.13 to 253.12 μg/kg, and the ratios of the compound from lesions and healthy parts were in the range of 7.61–12.59. Migration of FB_1_ and FB_2_ in sweet pepper was reported by Monbaliu et al. [19], and this is the first report about the diffusion of FA and MON in sweet pepper.

Mycotoxins can persist in infected plant tissues, and depending on physical or chemical properties (solubility, polarity, hydrophilicity, molecular weight, concentration, etc.) and tissue components, might also transfer from a rotten part of the plant tissues into the surrounding sound tissues, even in the absence of fungal growth. In this study, no NIV was detected in healthy pepper tissues maybe due to the low concentration, while BEA was not detected even at a high concentration (1019.60 μg/kg). Similar results were reported by Monbaliu et al. [19] that BEA can not be detected in surrounding tissues even with an extremely high concentration, 73,800 μg/kg, in pepper lesions. As shown in Figure 4, BEA is a cyclic hexadepsipeptide that contains three D-hydroxyisovaleryl and three N-methylphenylalanyl residues in an alternating sequence [35]. As an organic and non-polar compound, BEA is insoluble in the aqueous environment. Vegetable, and sweet peppers in particular, contain >90% water, which is probably why BEA was not detected in the surrounding tissues. These results demonstrated the possible risk of mycotoxin contamination in non-infected parts of food products, and some mycotoxins can diffuse into sound tissues. Since second-quality vegetables and fruits may be used to produce derivatives such as juices, jam, etc., further studies on migration behaviors and affecting factors of different mycotoxins in various vegetables and fruits are very important to establish suitable means of protecting consumers from exposure to toxic substances [20].

## 3. Conclusions

*Fusarium* species causing pepper fruit rot (external and internal) were analyzed in this study. Altogether, forty-two isolates belonging to *F. equiseti* (27 isolates), *F. solani* (10 isolates), and *F. fujikuroi* (five isolates) were identified with *F. equiseti* being the predominant species. To our best knowledge, this is the first report of *F. fujikuroi*, *F. equiseti* and *F. solani* associated with pepper fruit rot in China. Toxigenic profiles of ten pathogens were determined in sweet peppers, and six toxic metabolites (BEA, FA, FB_1_, FB_2_, MON, and NIV) were detected in total. Significantly different toxigenic profiles were observed among the three *Fusarium* species. Diffusions of FA, FB_1_, FB_2_, and MON from lesions into the surrounding sound tissues were observed in sweet peppers, and this is the first report about the migration of FA and MON in sweet peppers. Further studies on migration behaviors and affecting factors of different mycotoxins in various vegetables and fruit should be conducted.

## 4. Materials and Methods

### 4.1. Isolation and Purification of Fusarium Strains

During the growing season, pepper fruit samples were collected from different regions in China, including Hainan, Heilongjiang, Hunan, Shandong, Shanghai, and Zhejiang provinces (Table 1). Pepper fruits with visible symptoms (external or internal when cut open) were selected for pathogen isolation. Fungi were isolated using conventional methods as follows: Symptomatic tissues (3 × 3 cm) were surface-sterilized in 0.1% HgCl_2_ for 1 min, transferred into 70% ethanol for 30 s, then rinsed three times in sterilized distilled water, dried, and plated on 90 mm Petri dishes containing potato dextrose agar (PDA). After incubation for 3–5 days at 28 °C in the dark, colonies resembling morphologically to *Fusarium* were transferred onto new PDA. Plates were incubated at 28 °C in the dark until colonies developed, and then purified through serial transfers. No more than one strain per fruit was isolated. For each strain, a single spore culture was obtained by single-sporing as described before [36]. The pure cultures were used for the morphological and molecular characterization. Monoconidial strains were cryopreserved and maintained in tubes on PDA in the lab.

### 4.2. Nucleotide Maniplation

Mycelia plugs from 3-day-old PDA cultures were transferred to 50 mL of potato dextrose broth (PDB) medium and incubated with shaking (100 rpm) at 28 °C in the dark for 3 days. After incubation, mycelium was filtered through two-layered cheesecloth, washed with sterile water, then freeze-dried and ground to a fine powder using a TissueLyser II system (Qiagen Tissuelyser II, Retsch, Haan, Germany). Genomic DNA was extracted and purified using a Cetyl Trimethylammonium Bromide (CTAB) protocol as described before [37]. In brief, after homogenization, the ground power was suspended with 600 μL of CTAB lysis buffer, mixed well by shaking, and incubated at 65 °C for 1 h. After incubation, the solution was cooled at room temperature for 5 min, cellular debris were pelleted by centrifugation at 12,000 rpm for 10 min, and 500 μL of supernatant was transferred into a new tube. The supernatant was extracted with 500 μL of chloroform:isoamyl alcohol mixture (24:1, *v*/*v*). After centrifugation at 12,000 rpm for 10 min, 400 μL of aqueous phase was transferred to a fresh tube, then 400 μL of ice-cold isopropyl alcohol and 40 μL of sodium acetate (3 M, pH 5.2) were subsequently added to the samples. The tubes were mixed by gentle inversion. After incubation for 1 h at −20 °C, DNA was precipitated by centrifugation at 12,000 rpm for 10 min. The DNA sample was washed with 800 μL of pre-chilled 70% ethanol and air-dried before resuspension in 50 μL TE buffer (10 mM Tri-HCl, 0.1 mM EDTA, pH 8.0). DNA quantification and quality analysis were carried out by agarose gel electrophoresis with known DNA marker as standard.

### 4.3. Fusarium Strain Identification

The purified *Fusarium* strains were identified to species by morphological characteristics, and this was confirmed by *TEF-1α* gene sequence analysis of the representative strains. Methods for determining phenotypic characters and mycelial growth of *Fusarium* strains were from published protocols [21].

In order to verify the identity of *Fusarium* strains collected, DNA sequence comparisons were made for a subset of the strains using the *TEF-1α* gene, known as one of the most pertinent gene for determining the species rank in the *Fusarium* genus [23,24,25]. Portions of the *TEF-1α* gene were amplified with primer pair EF-1 (3′-ATGGGTAAGGA(A/G)GACAAGAC-5′) and EF-2 (3′-GGA(G/A)GTACCAGT(G/C)ATCATGTT-5′) in a thermal cycler. Polymerase chain reaction (PCR) was performed in a 50 μL reaction system afterwards [4], with minor modifications. PCR reaction mixtures contained 1× TransStar FastPfu Fly PCR SuperMix (TransGen Biotech, Beijing, China), 0.2 μM of each primer, and 50 ng of genomic DNA template. A negative control omitting the DNA template was used in every set of reactions. The thermal cycler (T100 Thermal Cycler, Bio-Rad, Hercules, CA, USA) conditions consisted of an initial denaturation step at 94 °C for 2 min, followed by 30 cycles of denaturation at 94 °C for 20 s, annealing at 58 °C for 20 s and extension at 72 °C for 30 s, then a final extension of 72 °C for 5 min. Amplified products (50 μL) were separated by electrophoresis on 1.5% (*w*/*v*) agarose gels. Gels were stained with ethidium bromide and photographed under UV light in the Bio-Imaging system (Bio-Rad, Hercules, CA USA). Fragments were excised and extracted from the gel using the QIAquick gel extraction kit (QIAGEN, Hilden, Germany) according to the manufacturer’s instructions. Purified amplicons were sequenced in both directions using an ABI3730XL DNA sequencer (Applied Biosystems, Foster City, CA, USA) for each strain, and contigs were assembled with Sequencher version 4.1 program (Gene Codes Corporation). The *TEF-1α* gene sequences generated in this study were subjected to similarity searches with the BLASTn network service of NCBI nucleotide database.

### 4.4. Phylogenetic Analysis

Portions of the *TEF-1α* gene sequences from eleven *Fusarium* strains were generated for phylogenetic analysis in this study (Table 1). All sequences were compared with sequences of *Fusarium* species available in the GenBank database through BLASTn searches for similar sequences.

Phylogenetic analyses were conducted using MEGA v. 5.10 [29] to characterize the genetic diversity and evolutionary relationships of the strains. *TEF-1α* sequences of twenty-five fungal strains belonging to *Fusarium* genus retrieved from the NCBI database were also used as references for constructing a phylogenetic tree. In total, thirty-six sequences were analyzed, including seven *F. equiseti* strains, ten *F. fujikuroi* strains, eight *F. solani* strains, three *F. concentricum* strains, two *F. lactisi* strains, two *F. proliferatum* strains, two *F. subglutinans* strains, and two *F. verticillioides* strains. The *TEF-1α* sequences of these *Fusarium* strains were all available in NCBI nucleotide database, and detailed information about their strain code, geographic origin, and host/substrate is listed in Table 3.

All sequences were aligned initially with ClustalX software [44] and the alignments manually edited. Phylogenetic analyses of the sequences were performed with MEGA5.1 for Neighbor-joining (NJ) analysis, and Kimura-2 parameter model and pairwise deletion option for gaps were used. The reliability of the tree topologies was evaluated using bootstrap support with 1000 pseudoreplicates of the data.

### 4.5. Mycotoxin Production Analysis in Pepper Fruits via LC-MS/MS

Fungal strains were initially grown on PDA in 90 mm diameter Petri dishes for 7 days at 28 °C in the dark, after which they were used to inoculate pepper fruits. Mature pepper fruits were inoculated with mycelium plug as described by Van Poucke et al. [4]. After incubation, the fruit tissues from different positions, including the inoculation site and healthy site, were collected, homogenized separately, and processed for mycotoxin detection analysis. Mycotoxin detection results from lesions were used to do the mycotoxin profile analysis, and the results from the healthy parts were used for the migration study.

For each sample, 2 g of the grounded material was extracted with 8 mL extraction solvent (acetonitrile:water = 84:16, *v/v*). Multi-component analysis of mycotoxins in the inoculated samples was performed as described previously [45,46]. In total, 36 mycotoxins were included in the multi-mycotoxin analyses: Aflatoxin B_1_ (AFB_1_), aflatoxin B_2_ (AFB_2_), aflatoxin G_1_ (AFG_1_), aflatoxin G_2_ (AFG_2_), aflatoxin M_2_ (AFM_2_), aflatoxin M_1_ (AFM_1_), beauvericin (BEA), citrinin (CIT), cyclopiazonic acid (CPA), deoxynivalenol (DON), 3-acetyldeoxynivalenol (3-ADON), 15-acetyldeoxynivalenol (15-ADON), deoxynivalenol-3-glucoside (DON-3G), deepoxydeoxynivalenol (Deep-DON), diacetoxyscirpenol (DAS), fusaric acid (FA), fumonisin B_1_ (FB_1_), fumonisin B_2_ (FB_2_), fusarenon-X (FUSX), gliotoxin, HT2 toxin (HT2), moniliformin (MON), neosolaniol (NEO), nivalenol (NIV), ochratoxin A (OTA), patulin (PAT), penitrem A (PenA), sterigmatocystin (SMC), T-2 toxin (T-2), verruculogen (VER), zearalenone (ZEN), α-zearalanol (α-ZOL), β-zearalanol (β-ZOL), α-zearalenol (α-ZAL), β-zearalenol (β-ZAL), and zearalanone (ZAN). HPLC or analytical grade of acetonitrile, hexane, and other chemical agents were purchased from Merck (Darmstadt, Germany). Deionized water purified by a Milli-Q water system (Millipore, Billerica, MA, USA) was used throughout the experiments.

## Figures and Tables

**Table:** 

Figure	R_1_	R_2_
Fumonisin B_1_	OH	OH
Fumonisin B_2_	OH	H
Fumonisin B_3_	H	OH

**Table 1:** *Fusarium* strains isolated in this study.

Species	Strain Code	Origin	Symptom (External/Internal)	GenBank Accession No.
*F. equiseti*	Q12001	Shanghai	external	-
	Q12002	Shanghai	internal	KF208617
	Q12003	Shanghai	external	KF208618
	Q12004	Shanghai	external	-
	Q12005	Shanghai	internal	KF208619
	Q12006	Shanghai	external	-
	Q12007	Shanghai	external	-
	Q12008	Shanghai	internal	-
	Q12009	Shanghai	external	-
	Q12010	Hainan	external	-
	Q12011	Shandong	external	-
	Q12012	Shandong	external	-
	Q12013	Shanghai	external	-
	Q12014	Shanghai	external	-
	Q12015	Shandong	external	-
	Q12016	Helongjiang	external	-
	Q12017	Zhejiang	external	-
	Q12018	Zhejiang	internal	-
	Q12019	Zhejiang	external	-
	Q12020	Zhejiang	external	-
	Q12021	Zhejiang	external	-
	Q12022	Zhejiang	internal	-
	Q12023	Zhejiang	external	-
	Q12024	Zhejiang	external	-
	Q12025	Hunan	external	-
	Q12026	Hunan	external	-
	Q12027	Hunan	external	-
*F. solani*	Q12028	Shanghai	external	-
	Q12029	Shanghai	external	KF208620
	Q12030	Shanghai	external	KF208621
	Q12031	Shanghai	external	-
	Q12032	Shanghai	external	-
	Q12033	Shanghai	external	-
	Q12034	Shanghai	internal	KF208622
	Q12035	Shanghai	internal	-
	Q12036	Shanghai	external	-
	Q12037	Shanghai	external	-
*F. fujikuroi*	Q12038	Hainan	external	KF208623
	Q12039	Zhejiang	external	KF208624
	Q12040	Zhejiang	external	KF208625
	Q12041	Hunan	internal	KF208626
	Q12042	Hunan	external	KF208627

**Table 2:** Mycotoxins produced by selected *Fusarium* strains in sweet pepper samples.

*Fusarium* Species	Strain Code	Position ^1^	Mycotoxins Detected (μg/kg)					
			BEA	FA	FB_1_	FB_2_	MON	NIV
***F. equiseti***	**Q12002**	IS	5.20	411.64	-	-	-	184.16
		HS	-	13.80	-	-	-	-
*F. equiseti*	Q12004	IS	9.12	160.32	-	-	-	-
		HS	-	-	-	-	-	-
*F. solani*	Q12029	IS	-	41.44	-	-	-	-
		HS	-	-	-	-	-	-
*F. solani*	Q12034	IS	98.48	78.98	-	-	-	-
		HS	-	-	-	-	-	-
*F. solani*	Q12037	IS	-	-	-	-	-	-
		HS	-	-	-	-	-	-
*F. fujikuroi*	Q12038	IS	153.84	10662.36	1512.48	1190.12	2439.48	-
		HS	-	592.00	45.36	31.67	193.75	-
*F. fujikuroi*	Q12039	IS	318.00	4870.56	1559.04	851.88	1429.76	-
		HS		130.40	41.08	24.30	172.13	-
*F. fujikuroi*	Q12040	IS	44.44	6522.72	43.64	26.96	151.72	-
		HS	-	874.04	-	-	-	-
*F. fujikuroi*	Q12041	IS	23.80	2940.72	3926.60	3734.16	1925.00	-
		HS	-	530.48	183.04	144.92	253.12	-
*F. concentricum*	MUCL54697	IS	1019.60	2886.08	-	-	35.80	-
		HS	-	55.64	-	-	-	-

^1^ IS, Inoculation site; HS, Healthy site.

**Table 3:** *Fusarium* strains used in phylogenetic analysis and their origins and GenBank accession numbers of *TEF-1α* genes.

Speices	Strain code	Origin	Host/Substrate	Accession number	Reference
*F. concentricum*	B2447B	Malaysia	Banana	KY379181	[38]
*F. concentricum*	MUCL54697	China	Sweet pepper	KC816735	[22]
*F. concentricum*	NRRL25202	Korea	Delphacidae	JF740760	[24]
*F. equiseti*	35b	China	Wheat	KY466715	-
*F. equiseti*	B1349T	China	Tomato	KM886212	-
*F. equiseti*	PAK54	USA	Loquat	KY523101	-
*F. equiseti*	Q12002	China	Sweet pepper	KF208617	This study
*F. equiseti*	Q12003	China	Sweet pepper	KF208618	This study
*F. equiseti*	Q12005	China	Sweet pepper	KF208619	This study
*F. equiseti*	TOR-9	Spain	Strawberry	KX215087	[39]
*F. fujikuroi*	37-EF1	Malaysia	Pineapple	KC584844	-
*F. fujikuroi*	BJ-1	China	Bletilla striata	MH263736	-
*F. fujikuroi*	F271	India	Rice	KM586385	-
*F. fujikuroi*	F326	India	Rice	KP009955	-
*F. fujikuroi*	M7055	China	Maize	KC964126	[40]
*F. fujikuroi*	Q12038	China	Sweet pepper	KF208623	This study
*F. fujikuroi*	Q12039	China	Sweet pepper	KF208624	This study
*F. fujikuroi*	Q12040	China	Sweet pepper	KF208625	This study
*F. fujikuroi*	Q12041	China	Sweet pepper	KF208626	This study
*F. fujikuroi*	Q12042	China	Sweet pepper	KF208627	This study
*F. lactisi*	NRRL25200	USA	*Ficus carica*	AF160272	[41]
*F. lactisi*	SPF22	Korea	Sweet pepper	JF411959	[5]
*F. proliferatum*	9174	Malaysia	Pitaya	JX869021	[42]
*F. proliferatum*	9194	Malaysia	Pitaya	JX869025	[42]
*F. solani*	DE17	Malaysia	Mangrove soil	KM096385	-
*F. solani*	N2211	Malaysia	Cucurbits	KT211623	-
*F. solani*	N2214	Malaysia	Cucurbits	KT211624	-
*F. solani*	NRRL52699	Colombia	Cercopidae	JF740782	[24]
*F. solani*	NRRL52704	USA	Tetranychidae	JF740786	[24]
*F. solani*	Q12029	China	Sweet pepper	KF208620	This study
*F. solani*	Q12030	China	Sweet pepper	KF208621	This study
*F. solani*	Q12034	China	Sweet pepper	KF208622	This study
*F. subglutinans*	MUCL52468	Belgium	Maize	HM067691	[25]
*F. subglutinans*	NRRL22016	USA	Maize	AF160289	[25,41,43]
*F. verticillioides*	ITEM2625	Slovakia	Maize	KF715264	-
*F. verticillioides*	NRRL22172	Germany	Maize	AF160262	[25,41,43]
